# Predicting Potential Suitable Habitats of Three Rare Wild Magnoliaceae Species (*Michelia crassipes*, *Lirianthe coco*, *Manglietia insignis*) Under Current and Future Climatic Scenarios Based on the Maxent Model

**DOI:** 10.3390/plants14040506

**Published:** 2025-02-07

**Authors:** Yu Fan, Weihao Yao, Zenghui Wang, Xinyue Fan, Shuyue Hu, Hongfei Wang, Jing Ou

**Affiliations:** College of Forestry, Guizhou University, Guiyang 550025, China; gzufanyu@163.com (Y.F.); ywh19831027397@163.com (W.Y.); 17385653247@163.com (Z.W.); xinyuefan1998@163.com (X.F.); 15180870742@163.com (S.H.); wanghongfei1719@163.com (H.W.)

**Keywords:** climate change, Magnoliaceae, suitable habitat, rare plant conservation, predictive model

## Abstract

In recent years, the impacts of climate change and human activities have intensified the loss and fragmentation of habitats for wild rare Magnoliaceae. Predicting the potential impacts of future climate change on the suitable habitat distribution of wild and endangered Magnoliaceae species is of great significance for their conservation and application. This study employs the optimized MaxEnt model to investigate current and future potential suitable habitats of three rare Magnoliaceae species (*Michelia crassipes*, *Lirianthe coco*, and *Manglietia insignis*). The dominant environmental variables influencing the distribution of three species were also explored. The results showed the following: (1) The potential habitat range of three Magnoliaceae species currently span from 92–122° N and 19–36° E. Variables associated with temperature (bio2, bio9, bio4) and altitude (Ele) significantly influence the distribution of these species, with precipitation (bio17) and ultraviolet radiation (UVB4) playing a minor role. The warm and humid climate in central and southern China is highly conducive to their growth. (2) Under the SSP126 scenario, after the mid-21st century, the suitable habitat area of *Michelia crassipes* has undergone a fluctuating trend of initial increase followed by decrease, reducing to 51.84 × 10^4^ km^2^ in 2090. On the other hand, both the suitable habitat areas of *Lirianthe coco* and *Manglietia insignis* show an upward trend. Under the SSP245 and SSP585 scenarios, the total suitable habitat areas of these three rare Magnoliaceae species gradually decrease. (3) We compared the priority protection areas with existing Protected Areas (PAs) in gap analysis; 96.84% of priority conservation areas are lacking effective protection. (4) The distribution centroid is constantly moving to western China. In order to address habitat fragmentation, it is recommended that the range of natural reserves be expanded and ecological corridors be established in the future, preferably according to the predicted suitable climate for protected areas and refuges or habitats for these species. Overall, these findings provide valuable insights for the preservation, stewardship, and utilization of the endangered species of Magnoliaceae under the circumstances of projected global climate change.

## 1. Introduction

The current greenhouse gas concentration is continuously rising, and the global surface temperature is increasing. Over the past 50 years, the loss of biodiversity has been intensifying [1,2]. A quarter of global plant species will face extinction [3]. Numerous rare plant habitats have been destroyed, leading to a sharp decline in their populations [4]. Climate change is considered by many scholars to be a key influence on the geographical distribution of plants [5], and exploring the relationship between plants and climatic variables has become a hot issue in biogeography and global climate change research [6,7]. According to data from the Intergovernmental Panel on Climate Change Sixth Assessment Report (IPCC AR6), global temperatures are projected to rise by 1.5 °C by 2040, which will result in significant changes in the geographical distribution patterns of most plants [8,9]. Some species are experiencing shrinking habitats and are even facing extinction in the wild [10,11], causing a huge impact on global biodiversity conservation [12]. Therefore, under the context of climate change and frequent extreme weather events, scientifically predicting the distribution areas of plants and implementing in situ and ex situ conservation measures are both of great importance for biodiversity conservation [13].

Ecological Niche Models (ENMs) are computational models used in ecology and conservation biology to predict the potential distribution and habitat suitability of species, which use data on the species distributions, as well as environmental variables such as temperature, precipitation, and soil, to identify the environmental variables that contribute to the species’ distribution pattern [14]. We analyze these relationships to further research the response of species migration to climate change. Common niche models include the CLIMEX model, Genetic Algorithm for Ruleset Production (GARP) [15], Random Forest (RF) [16], and the MIGCLIM model [17]. The MaxEnt model is the most widely used and can more accurately identify the potential habitat area of a species [18]. Currently, MaxEnt has been used to predict potential habitats for plants and animals [19], for endangered species protection [2], and to prevent and manage the spread of invasive alien species [20], as well as for pest control [21].

Magnoliaceae is a family of flowering plants, mostly tall trees, which are known to the public for their showy fragrant flowers. This family is valued for both ornamental and medicinal purposes and is cultivated worldwide. Magnoliaceae are considered one of the basal groups of dicotyledonous plants because they share many primitive characteristics with early angiosperms [22]. There are 15 genera and about 330 species in the world and 11 genera and more than 160 species in China, mainly in tropical and subtropical areas of south-eastern Asia [23]. Various studies have also confirmed that China is highly likely to be the origin of Magnoliaceae [24]. In recent years, due to reasons such as excessive exploitation, habitat loss, and climate change, Magnoliaceae have become one of the most threatened families [25]. Previous research on Magnoliaceae has mostly focused on their morphological features [26,27], taxonomy [24], and endangered status [28]. Only a handful of studies have focused on changes in its range in China [29,30]. By studying the current and future potential distribution ranges on a large scale and screening the main environmental variables affecting the distribution of Magnoliaceae, we can identify suitable areas for introduction and provide scientific references for their conservation and application.

In this study, we utilized MaxEnt, along with ArcGIS software to investigate three rare Magnoliaceae species that are included in the Redlist of China’s Biodiversity (*Michelia crassipes*, *Lirianthe coco*, and *Manglietia insignis*). Based on the actual distribution data of plants and relevant environmental variables, we predicted the potential changes in suitable habitats of these species under current and future climate change scenarios. The main objectives of this research are as follows: (1) predicting the potential suitability habitat of the three Magnoliaceae species under current climatic scenario; (2) identifying the key environmental variables that influence the potential distributions of the three species; (3) identifying the conservation gaps; (4) analyzing the future distribution of potentially suitable habitats for the three plant species under different climatic scenarios. The results of the study can provide a scientific basis and reference for relevant departments in the investigation of wild Magnoliaceae resources and the formulation of targeted conservation programs, and provide a reference basis for the conservation, management, development, and utilization of wild Magnoliaceae in China.

## 2. Result and Analysis

### 2.1. Model Accuracy Evaluation and Contribution of Environmental Variables

**Figure 1:**
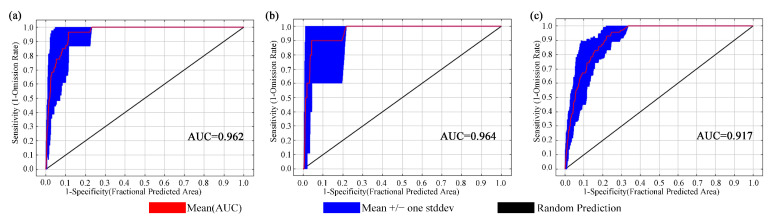
Under the current climatic conditions (1970–2000), the ROC curves and AUC values of three Magnoliaceae after running the experiment 10 times are as follows: (**a**) *M. crassipes*, (**b**) *L. coco*, (**c**) *M. insignis*. The closer the AUC value is to 1, the better the predictive effect of the model.

With the default parameter settings of the MaxEnt model, the value of delta AICc is 1236.299. However, when the model was established with the optimized parameters (FC = LQ and RM = 2), a delta AICc value of 0 was obtained. The optimized parameters were used to simulate the appropriate spatial distribution of three Magnoliaceae species in China. Under this model, the average AUC for 10 repetitions for *M. crassipes*, *L. coco*, and *M. insignis* were 0.962, 0.964, and 0.917, respectively, which indicated an excellent performance of the models (Figure 1).

**Figure 2:**
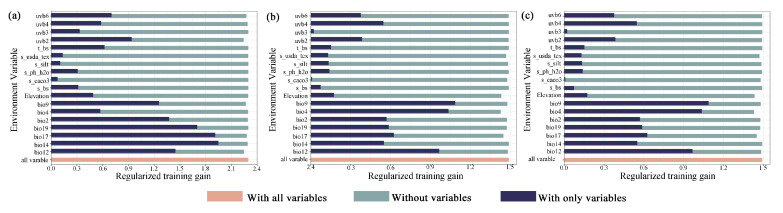
The importance of environmental variables evaluated by Jackknife testing: (**a**) *M. crassipes*, (**b**) *L. coco*, (**c**) *M. insignis*. The length of the dark blue bar reflects the degree to which the environmental variable influences the distribution of the species; longer bars indicate a greater impact.

The dominant environmental variables affecting the habitat distribution of the three Magnoliaceae species can be identified by the percentage contribution in the predictions and Jackknife test results (Figure 2). The percentage contribution values (Table 1) are average values established over 10 replicate runs; we identified the four environmental variables with the highest contribution rate as the dominant environmental variables affecting the potential distribution of three Magnoliaceae species, and their response curves with single environmental variables were derived by modeling.

#### 2.1.1. The Dominant Environmental Variables for the Distribution of *M. crassipes*

**Figure 3:**
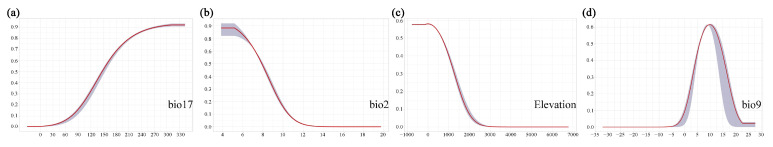
Response curves of major environmental variables of *M. crassipes*. The relationship between major environmental variables (X-axis) and the probability of the *M. crassipes* distribution (Y-axis) The red lines represent the mean, while the light grey borders represent the standard deviation for 10 replications.

For *M. crassipes*, precipitation in the driest quarter (bio17, with 70.8% contribution) was the most influential environmental variable. Meanwhile, mean of monthly (max temp–min temp) (bio2, with 5.5% contribution), elevation (Ele, with 3.8% contribution), and the cumulative contribution of mean temperature in the driest quarter (bio9, with 3.7% contribution) were also influential variables. By utilizing the response curves (Figure 3), we have observed that the probabilities of the presence of *M. crassipes* will continue to increase with increasing precipitation in the driest quarter (bio17). Additionally, the mean of monthly (max temp–min temp) (bio2) is negatively correlated with the probabilities of the presence of *M. crassipes*, which is nearly consistent with the response curve trend of elevation (Ele). When the cumulative contribution of mean temperature in the driest quarter (bio9) was below 8.43 °C, it was positively correlated with the probabilities of the presence of *M. crassipes*. When bio9 was above 8.43 °C, the probabilities of the presence of *M. crassipes* would continuously decrease with the increase of bio9. When the temperature rose to 18.35 °C, the probabilities of the presence dropped to below 0.2; at this temperature, *M. crassipes* cannot grow normally. Therefore, the most suitable habitat conditions for the species are as follows: bio17 ranged from 163.49 to 343.20 mm, bio2 ranged from 3.83 to 7.21 °C, elevation is about 400 m, and bio9 ranged from 8.43 to 11.30 °C.

#### 2.1.2. The Dominant Environmental Variables for the Distribution of *L. coco*

**Figure 4:**
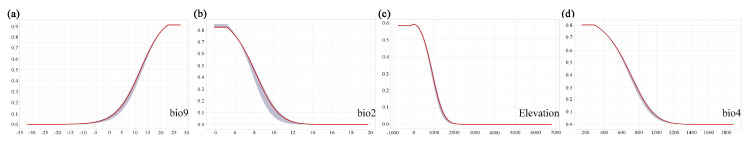
Response curves of major environmental variables of *L. coco.* The relationship between major environmental variables (X-axis) and the probability of the *L. coco* distribution (Y-axis) The red lines represent the mean, while the light grey borders represent the standard deviation for 10 replications.

The Magnoliaceae family exhibits a wide distribution in tropical and subtropical regions and is susceptible to temperature variations. We analyzed the impact of environmental variables on *L. coco* distribution by creating response curves (Figure 4). The result showed that the cumulative contribution of mean temperature in the driest quarter (bio9, with 34.8% contribution) and mean diurnal range (mean of monthly (max temp–min temp)) (bio2, with 23.4% contribution) was more than 58%, rising to over 70% with the inclusion of elevation (Ele, with 14% contribution). Due to the limitation of temperature, the probabilities of the presence of *L. coco* shows an obvious negative correlation with elevation. Temperature seasonality (standard deviation × 100) (bio4), which explained 11.3% of the variation, was also an influential variable for *L. coco*. Optimal habitat conditions for *L. coco* are as follows: bio9 ranged from 13.93 to 26.67 °C, bio2 ranged from 3.83 to 7.17 °C, elevation was about 500 m, and bio4 ranged from 140.49 to 555.67.

#### 2.1.3. The Dominant Environmental Variables for the Distribution of *M. insignis*

**Figure 5:**
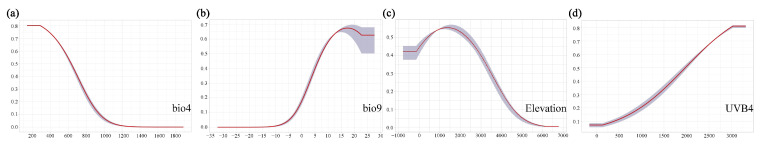
Response curves of major environmental variables of *M. insignis*. The relationship between major environmental variables (X-axis) and the probability of the *M. insignis* distribution (Y-axis) The red lines represent the mean, while the light grey borders represent the standard deviation for 10 replications.

The four dominant environmental variables affecting *M insignis* distribution are temperature seasonality (standard deviation × 100) (bio4, with 42.1% contribution), mean temperature in the driest quarter (bio9, with 30.5% contribution), elevation (Ele, with 6.1% contribution), and mean UV-B of lowest month (UVB4, with 4.6% contribution). Among them, bio4 and bio9 have a greater impact on the distribution of *M insignis*, with their percentage contribution being as high as 66.9%. When bio4 is below 140.49–555.67, *M insignis* has high probabilities of presence. As bio4 continues to increase, the distribution probability of *M insignis* gradually decreases. There is a positive correlation between bio9 and the probabilities of the presence of *M insignis*. When bio9 reaches 22.41 °C, the distribution probability of M insignis begins to decrease slowly, stabilizes when it drops to 22.77 °C, and remains above 0.6. The probability of the presence of *M insignis* is positively correlated with an altitude below 1629.33 m and continues to decrease with the increase in altitude after exceeding 1629.33 m (Figure 5). The habitat suitability of *M insignis* increased with the increase in mean UV-B of the lowest month (UVB4), and the probability of the presence reached a peak and stabilized after UVB4 increased to 3031.28 jm^−2^.day^−1^. Therefore, when bio4 is 140.49–555.67, bio9 is between 10.82 and 27.67 °C, the altitude is 1114.6–1629.33 m, and UVB4 is 2267.66–3315.91 jm^−2^.day^−1^, the environment is the most suitable for *M insignis* growth.

### 2.2. Potential Distribution Under Current Climate Conditions

**Figure 6:**
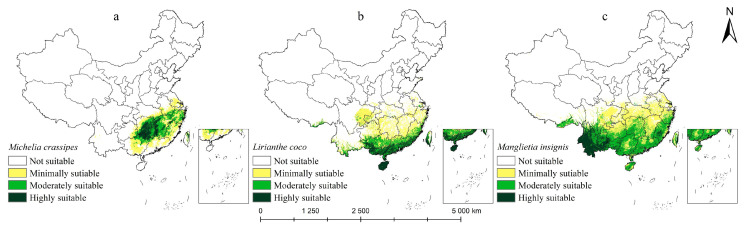
Current distribution of Magnoliaceae in China: (**a**) *M. crassipes*, (**b**) *L. coco*, (**c**) *M. insignis*.

**Figure 7:**
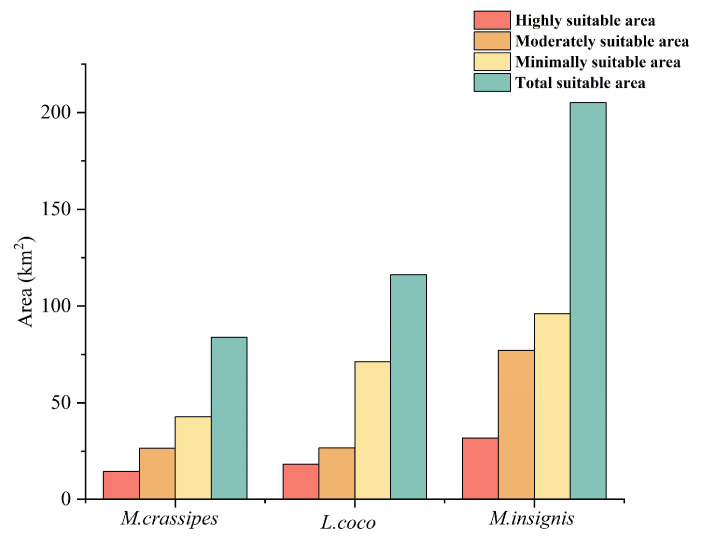
Suitable habitat areas for three Magnoliaceae species.

According to the model simulation, the most suitable habitats of the three rare Magnoliaceae species are distributed in the southern part of China (Figure 6). This further proves that southern China is probably the modern distribution center, differentiation center, and preservation center of Magnoliaceae. After calculating the area of different suitable areas (Figure 7), we found that the total suitable area of *M. crassipes* was 83.96 × 10^4^ km^2^, mainly distributed in southeast China, and a small amount in Yunnan-Guizhou, where the most highly suitable area was 14.57 × 10^4^ km^2^, concentrated in Hunan, west Jiangxi, and northeast Guangxi, and it continues to spread centered on the highly suitable areas in Hunan and Jiangxi. As a whole, the distribution pattern of the suitable areas is that the moderately and minimally suitable areas surround the periphery of the highly suitable areas. The total suitable area of *L. coco* was 116.22 × 10^4^ km^2^, of which the highly suitable area was 18.33×10^4^ km^2^, accounting for 1.91% of the total land area of China. This was concentrated in the southern coastal provinces. The habitat suitability of *L. coco* decreases northward from Guangxi, Guangdong, Fujian, and Hainan. The distribution area of *M. insignis* is much higher than that of *M. crassipes* and *L. coco*, accounting for 21.36% of China’s land area (205.04 × 10^4^ km^2^), and the highly suitable area is concentrated in southwest Yunnan, Fujian, Guangxi, and Xizang, with a total distribution area of 31.87 × 10^4^ km^2^. The characteristics of narrow habitat distribution, severe fragmentation, and the small area of highly suitable areas not only reflect the endangered status of Magnoliaceae but also reveal the reasons for the difficulty in protecting Magnoliaceae.

### 2.3. Comparison of the Geographical Distribution and Ecological Niche

We used ENMTools to calculate the niche breadth and niche and range overlap of three Magnoliaceae species, and the threshold for range distribution was set to 0.4. According to Table 1, under the current climate scenario, the niche breadth of *M. insignis* had the highest among of the three species (B_2_ = 0.913), and the niche overlap of the three species was higher than 0.5, among which *L. coco* and *M. insignis* had the highest niche overlap (D = 0.639). The range overlap of *L. coco* and *M. insignis* was 0.780, but the range overlap of *L. coco* and *M. crassipes* was low (0.059).

**Table 1:** Niche breadth and niche and range overlap of three Magnoliaceae.

Niche (Above the Diagonal)\Range Overlap (Below the Diagonal)	*M. crassipes*	*L. coco*	*M. insignis*	B_2_
*M. crassipes*	1	0.520	0.524	0.862
*L. coco*	0.059	1	0.639	0.879
*M. insignis*	0.522	0.780	1	0.913

### 2.4. Conservation Gaps

**Figure 8:**
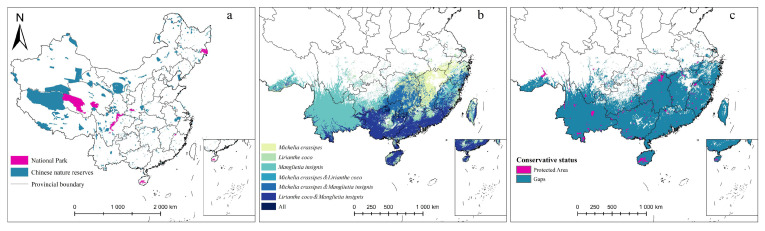
Protected natural areas in China (**a**), status of coexisting distribution of three Magnoliaceae (**b**), and protected area and gap areas of three Magnoliaceae (**c**).

By overlapping the location of the three rare Magnoliaceae priority protection areas with the range of PAs (Figure 8), we found the three species of wild Magnoliaceae currently occupy 54.73 × 10^4^ km^2^ of priority reserves in China, but with only 1.73 × 10^4^ km^2^ distribution coverage by natural protected area, with over 96.84% of the area not protected. The protection areas of the National Park of Hainan tropical rainforest and southern Yunnan are concentrated, and the PAs of Guangxi and Fujian are scattered. The three species of wild Magnoliaceae under the coverage area have been effectively protected, but there is still a large area of conservation gaps in Guangdong and Jiangxi.

### 2.5. Potential Distribution of Three Magnoliaceae Under Future Climate Conditions

#### 2.5.1. Potential Habitat for *M. crassipes* Under Climate Change Scenarios

**Figure 9:**
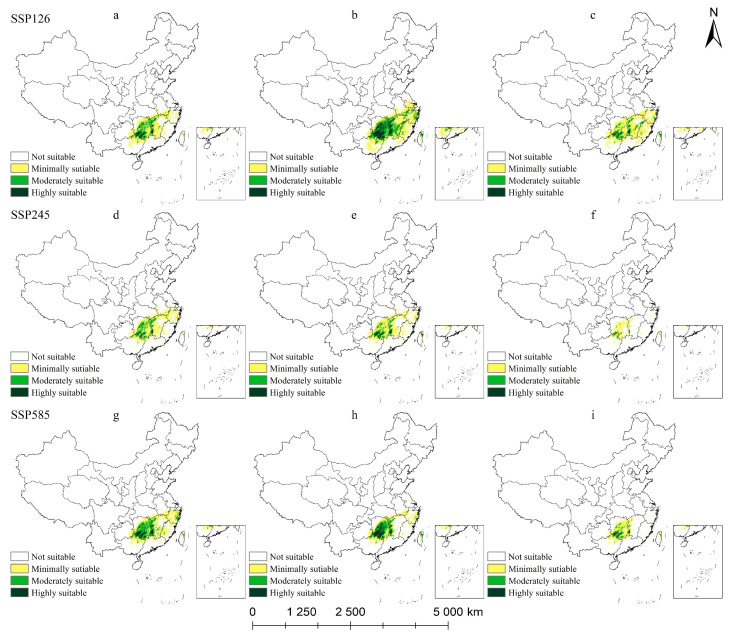
Potential suitable habitat of *M. crassipes* in China under future climate scenarios: (**a**) SSP126-50, (**b**) SSP126-70, (**c**) SSP126-90, (**d**) SSP245-50, (**e**) SSP245-70, (**f**) SSP245-90, (**g**) SSP585-50, (**h**) SSP585-70, (**i**) SSP585-90.

It can be seen from Table 2 and Figure 9 that the suitable area of *M. crassipes* will change obviously under the future climate scenario. By the 2050s, under the SSP126 climate scenario, the total suitable area for *M. crassipes* decreased by 37.93 × 10^4^ km^2^. By the 2070s, the suitable area increased to 72.14 × 10^4^ km^2^ but then dropped sharply to 20.31 × 10^4^ km^2^ in the 2090s. The overall trend was decrease–increase–decrease. Under the SSP245 climate scenario, the suitable area for *M. crassipes* changed by 4.07 × 10^4^ km^2^ between the 2050s and the 2070s, with the smallest change rate, indicating that climate change in these two periods under this scenario had little impact on the distribution of suitable areas for *M. crassipes*. However, in the 2090s, the suitable area dropped to 13.12 × 10^4^ km^2^, and the contraction rate was as high as 65.46%. The contraction areas were concentrated in Jiangxi, Fujian, Zhejiang, and other places. Under the SSP585 climate scenario, the total suitable habitat area of *M. crassipes* decreased year by year; during the study period, a total of 62.63 × 10^4^ km^2^ was lost. With the high intensity of human activities, the habitat suitability of *M. crassipes* continuously decreased, and the moderately and highly suitable areas were gradually degraded into minimally or not suitable areas. By using MaxEnt to predict the future climate change scenario of *M. crassipes*, we found that, under different climate scenarios, the habitat suitability of *M. crassipes* decreased continuously, and the suitable habitat was narrower. The spatial pattern changes of total suitable areas were shrinking to the central region of Hunan, as warm and humid climates provide shelter for *M. crassipes*.

#### 2.5.2. Potential Habitat for *L. coco* Under Climate Change Scenarios

**Figure 10:**
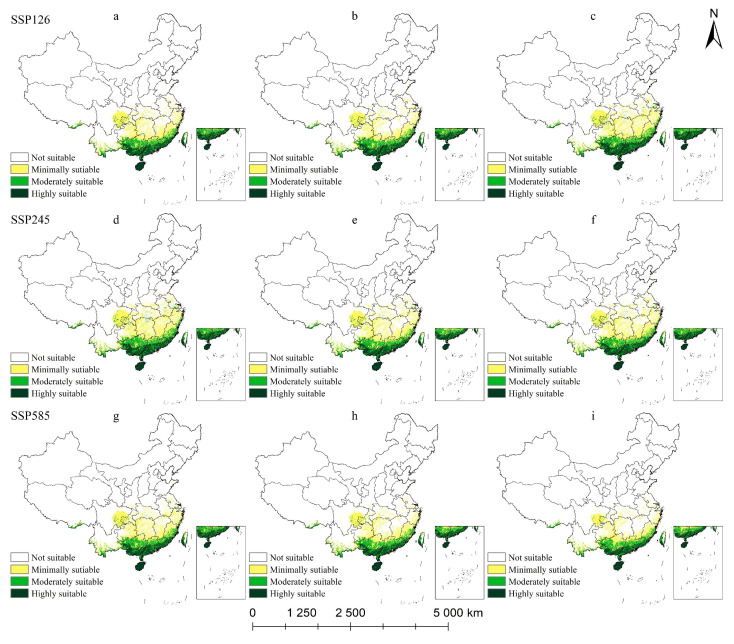
Potential suitable habitat of *L. coco* in China under future climate scenarios:(**a**) SSP126-50, (**b**) SSP126-70, (**c**) SSP126-90, (**d**) SSP245-50, (**e**) SSP245-70, (**f**) SSP245-90, (**g**) SSP585-50, (**h**) SSP585-70, (**i**) SSP585-90.

The distribution patterns of suitable areas for *L. coco* are very different under different shared socioeconomic paths (Table 3, Figure 10). The SSP126 climate scenario is a climate model for green growth that emphasizes inclusive development that respects the environment. Under this climate scenario, the total suitable area for *L. coco* first decreased and then increased; by the end of the 2070s, the total suitable area decreased by 17.55 × 10^4^ km^2^, with a change rate of −15.10%, but the highly suitable area increased slowly with time. Between the 2070s and 2090s, the total suitable area increased to 119.86 × 10^4^ km^2^, which is an expansion of 3.64 × 10^4^ km^2^ compared with the current total suitable area; this showed that it is more suitable for *L. coco* growth under the low-carbon path of human cooperation. Under the SSP245 climate scenario, the total suitable area for *L. coco* peaked in the 2050s (125.54 × 10^4^ km^2^) and then began to shrink; the suitable area of *L. coco* changed little under this scenario. Under the SSP585 climate scenario, the suitable area of *L. coco* decreased year by year, and most of the changes occurred in the minimally suitable area, which decreased by 21.54 × 10^4^ km^2^ during the study period. Under different climate scenarios (SSP126 and SSP245), the suitable area of *L. coco* showed a fluctuation pattern, and the overall change is relatively smooth, but under the SSP585 climate scenario there is a large amount of loss of suitable area in the Sichuan basin and the Hunan–Jiangxi border area.

#### 2.5.3. Potential Habitat for *M. insignis* Under Climate Change Scenarios

**Figure 11:**
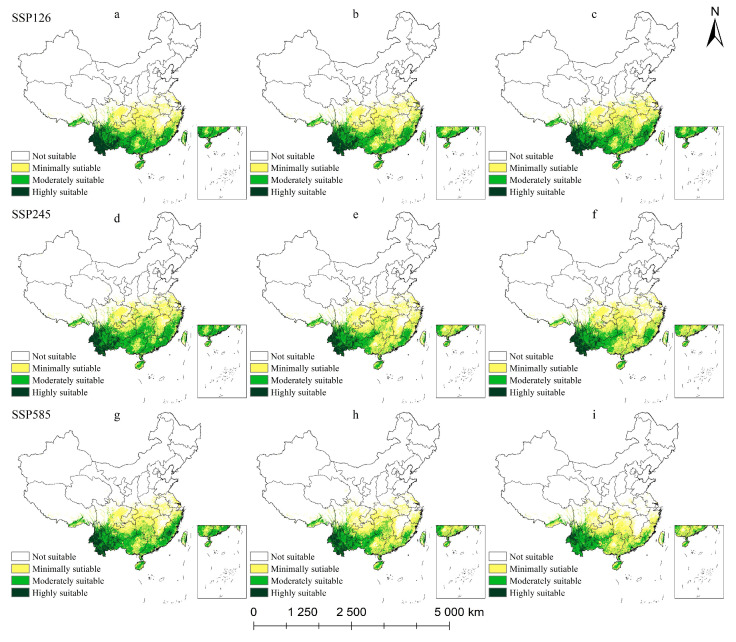
Potential suitable habitat of *M. insignis* in China under future climate scenarios: (**a**) SSP126-50, (**b**) SSP126-70, (**c**) SSP126-90, (**d**) SSP245-50, (**e**) SSP245-70, (**f**) SSP245-90, (**g**) SSP585-50, (**h**) SSP585-70, (**i**) SSP585-90.

The total suitable area for *M. insignis* exceeds that of the other two Magnoliaceae species (Table 4 and Figure 11). Under the three future climate scenarios, the suitable areas of *M. insignis* changed to different degrees. Under the SSP126 climate scenario, the total suitable area of *M. insignis* increased to 202.58 × 10^4^ km^2^ at the end of the 21st century. The total suitable area based on the SSP245 scenario of *M. insignis* decreased in the 2070s (18.46 × 10^4^ km^2^), and the highly suitable area was reduced by 4.81 × 10^4^ km^2^. Under the SSP585 climate scenario, the total suitable area for *M. insignis* decreased continuously. The habitat suitability in southwestern Yunnan was continuously degraded, the fragmentation trend accelerated, which is most severe in highly suitable areas. At the end of the 21st century, the total suitable area lost 54.11 × 10^4^ km^2^ compared with the current level, and the area of moderately and highly suitable areas decreased by 52.18 × 10^4^ km^2^, accounting for 96.42% of the total lost area.

### 2.6. Centroid Shifts in Direction and Distance of Different Species

**Figure 12:**
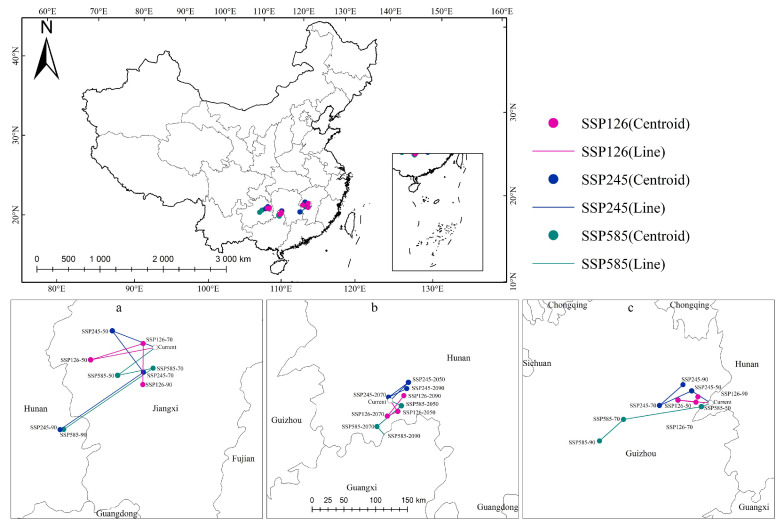
Total suitable habitat centroid distribution shifts for three Magnoliaceae under climate change: (**a**) *M. crassipes*, (**b**) *L. coco*, (**c**) *M. insignis*).

Changes in species distribution patterns are the most intuitive reflection of climate change [31]; the distribution centroid of all plants will migrate in different directions and to different distances over time [32]. Figure 12 shows that the centroid migration of the three Magnoliaceae species was mainly concentrated in Guizhou, Hunan, and Jiangxi (Table 5).

Under the SSP126 climate scenario, the centroid of suitable habitat for *M. crassipes* moved southwestward before moving northwest of the current distribution in the 2070s (115.08° E, 26.96° N) and shifted 64.70 km north to Ji’an City, Jiangxi Province (114.99° E, 26.96° N). Under the remaining two climate scenarios (SSP245 and SSP585), the centroid moved to the southwest and northwest, respectively, and then migrated to Zhuzhou, Hunan Province.

For *L. coco*, the centroid transfer changed little for the suitable habitat under the different climate scenarios; it mainly changed between Wugang City, Chengbu County, and Xining County in Hunan Province. The centroid of suitable habitat under the SSP126 and SSP245 climate scenarios moved to northwest, and under the SSP585 climate scenario the centroid migrated southward from the current centroid (110.52° E, 26.61° N) to Guilin, Guangxi (110.39° E, 26.14° N). Additionally, the centroid of suitable habitat under the SSP245 moved the longest distance; the migration was 40.58 km, 38.86 km, and 31.53 km, and all occurred near Wugang city.

The centroid of suitable habitat for *M. insignis* changed less under the SSP126 climate scenario, but under the SSP245 climate scenario, affected by the significant reduction in the habitable area of Guangxi and Guangdong, the current centroid (109.10° E, 27.28° N) shifted from northwest (108.81° E, 27.47° N) to southwest and then to northeast (108.68° E, 27.56° N), from Huaihua City in Hunan Province to Tongren City in Guizhou Province. Under the SSP585 climate scenario, as the climate warms, the centroid of M. insignis continues to shift southwestward, and the centroid shifted 124.00 km between the 2050s (108.95° E, 27.24° N) and the 2070s (107.73° E, 27.11° N), and then 50.82 km to 107.34° E, 26.82° N (2090s).

## 3. Discussion

### 3.1. Model Accuracy Analysis

The limited environmental variables cannot fully reflect the complex process of the evolution of geographical distribution of species [14], and the selection of environmental variables and overfitting of distribution points will affect the accuracy of the model. In this study, we eliminated the environmental variables with low contribution rates and redundant distribution points by Pearson correlation analysis and ENMTools. According to previous research, Magnoliaceae are mainly distributed in an area of 500–1500 m in southern China [11], basically consistent with the predicted results of this study. We used the ENMeval package in R 4.3.2 to select the optimal model tuning parameters for each species: the selected parameters of the delta. The AICc value is 0, and the corresponding RM and FC adjust the parameters for the optimal model. The AUC values of the three Magnoliaceae were all greater than 0.9, and the prediction effect was excellent, which further confirmed the authenticity and reliability of the prediction results.

### 3.2. The Predominant Environmental Variables Influencing Different Magnoliaceae Species

The dominant environmental variables affecting three Magnoliaceae species were screened by Jackknife and Pearson correlation as the difference of dominant environmental variables also reflects their preference for different habitat environments. In this study, bio9 and elevation affected the distribution of all three Magnoliaceae species, but different species had different preferences for bio9. While bio9 was suitable for *M. crassipes* growth in the range of 8.43–11.30 °C, *L. coco* and *M. insignis* were most suitable for growth in the range of 15–25 °C. Elevation is a combined response of air temperature and moisture variables. The suitable altitude for *M. crassipes* and *L. coco* is between 400 and 500 m. Affected by UVB4, the optimal growth altitude of *M. insignis* is more than 1100 m; the ultraviolet radiation in low-latitude, high-altitude regions is more suitable for its growth [33,34]. The difference in altitudinal distribution may be one of the main reasons for the potential geographical distribution differences among the three Magnoliaceae species.

The distribution of Magnoliaceae was significantly affected by temperature variables. The probability of the presence of some Magnoliaceae plants decreases when mean temperature in the driest quarter (bio2) exceeds 8 °C [35]; areas with bio2 of 3.8 to 7.2 °C are more suitable for *M. crassipes* and *L. coco* growth and have a high probability of presence, which is consistent with previous studies. The percentage contribution of bio4 to *L. coco* and *M. insignis* also proves that the need for a warm environment is one of the main factors restricting the spread of some Magnoliaceae plants to the northern high latitudes; thus, the total suitable area was reduced. Adequate precipitation can prevent or alleviate drought effects and supply plant seeds with the necessary water to sustain vitality during dormancy [36], which in turn ensures the continuation of the species [37]. Among the three Magnoliaceae species, *M. crassipes* had the most obvious habitat suitability response to water change; the percentage contribution of bio17 to the distribution of its potential suitable areas was as high as 70.8%. When the precipitation in the driest quarter (bio17) ranged from 163.49 to 343.20 mm under the environment of existence, probability is higher. Precipitation has the greatest impact on the distribution of *M. crassipes*, which is consistent with previous studies [38]. In the context of climate change, the interplay of environmental variables, including temperature, altitude, and precipitation, influences the habitat migration patterns of the majority of Magnoliaceae species [29,39,40].

### 3.3. Suitable Habitat and Its Dynamics Change

This study predicts the suitable habitat areas for three seriously threatened Magnoliaceae species, and we found that, under the current climate scenario, the niche widths of the three plants are *M. insignis* (B_2_ = 0.913) > *L. coco* (B_2_ = 0.879) > *M. crassipes* (B_2_ = 0.862). This shows that *M. insignis* is the most widely distributed, which is consistent with the predicted suitable area, and the niche overlap was all higher than 0.5, which also indicated that the three Magnoliaceae species had similar responses to environmental variables.

The potential suitable areas of three rare Magnoliaceae species were predicted based on the MaxEnt model. We found that, under the SSP126 scenario, the total suitable area for *M. crassipes* showed an obvious downward trend after increasing in 2070. The overall trend of the total suitable area for *L. coco* and *M. insignis* under this path was to first decrease and then increase. There is an upward trend after the middle of the century, indicating that, under the SSP126 scenario [41], human beings’ active environmental protection measures can promote the spread of suitable plant areas to a certain extent [42]. However, under the SSP245 and SSP585 scenarios, the potential suitable habitats of *M. crassipes*, *L. coco*, and *M. insignis* were reduced to different degrees with time, and the highly suitable areas were degraded to the moderately suitable or minimally suitable areas, consistent with previous research results [10]. The high-concentration shared socioeconomic path showed a more pronounced decreasing trend in all periods compared to the low-concentration path. Because of its narrow niche width, *M. crassipes* is the most sensitive to the change of hydrothermal conditions, and the area change of its potential suitable area is the most significant reduction under the background of climate change [43]. This is similar to previous findings that Magnoliaceae communities in eastern China are more vulnerable to environmental changes [44].

Magnoliaceae plants are mostly distributed in tropical and subtropical regions, and the increase in temperature will promote the expansion of some species to northern high latitudes [29]. Shen et al. (2022) analyzed the differences in the distribution of suitable habitats for the two species of Liriodendron and found that Magnoliaceae such as *Liriodendron tulipifera*, *Oyii* [12], *Yulania denudata*, *Yulania sprengeri*, and *Yulania biondii* were similar [39]. They will continue to spread to high latitudes and high altitudes in the context of future climate change. In contrast, suitable habitats for *Liriodendron chinense* show a localized contraction to the north in the future, suggesting that the global warming context is not beneficial for habitat dispersal for all plants [37]. The continuous migration of *M. crassipes*, *L. coco*, and *M. insignis* to the southwest and northwest suggests that the different adaptive capacities of different species in the same family to future climate change are the main reason for the differences in migration changes in the suitable areas [45]. The loss of suitable habitat and the serious habitat fragmentation problem indicates that the adaptation mechanism of the three Magnoliaceae species is not sustainable with the increasing temperature of the earth’s surface [14]. Habitat fragmentation restricts the exchange of plant populations and gene flow, leading to population isolation and a subsequent reduction in genetic diversity [46]. The distribution centers of all three Magnoliaceae species shifted to lower latitudes; limited by hydrothermal conditions, the centroids of the three Magnoliaceae species mainly move in the subtropical monsoon climate zone. The results also confirm that the species diversity of Magnoliaceae plants in east Asian subtropical Evergreen Broad-Leaf Forest (EBLF) regions has not exhibited significant changes over different time periods [44]. It can be used as a refuge for the long-term conservation of Magnoliaceae diversity.

### 3.4. Endangered Status and Conservation Recommendations

The prediction results of this study show that the current potential suitable areas of the three Magnoliaceae plants are more extensive than the actual distribution, but only 3% of the suitable areas are currently protected, and there is a large number of conservation gaps, like other endangered and rare plants [2]. From a long-term perspective, the conservation prospects of the three magnolia species are not positive. This study suggests that overlapping areas covering moderately and highly suitable areas of more than two Magnoliaceae species should be designated as priority conservation areas for three rare Magnoliaceae species, including most areas of Guangxi and Hainan, central and southern Hunan, southern Guangdong, Fujian and Zhejiang coastal city clusters, and local areas of southern Yunnan and Jiangxi. The priority protection areas delineated based on the research results involve two global biodiversity hotspots: the Indo-Burma and the mountains of southwest China [47]. The area has significant biodiversity, with natural conditions consistent with the growth habits of most Magnoliaceae [11], but is vulnerable to human activities. Conservation policies should be developed to limit human disturbance of Magnoliaceae habitat. It is necessary to expand existing PAs and establish an ecological corridor [48]. We recommend field investigation in priority protection areas, including mapping the actual distribution and population dynamics of the three species of Magnoliaceae plants, and establishing long-term monitoring projects [49], which will increase the understanding of the ecological and genetic characteristics of wild endangered Magnoliaceae species [50]. These studies will be conducive to protection and can provide scientific reference useful for the domestication and industrial usage of Magnoliaceae.

## 4. Materials and Methods

### 4.1. Species Data Sources

**Figure 13:**
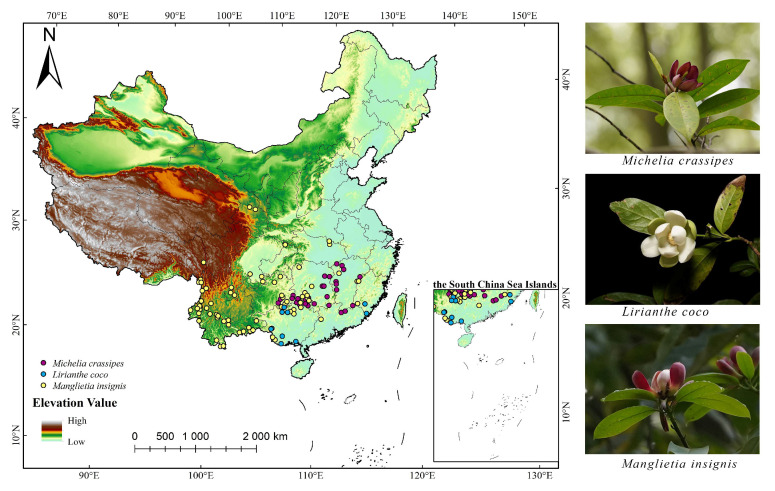
Distribution sites of three Magnoliaceae species in China (*M. crassipes*, *L. coco*, *M. insignis*).

The three species wild occurrence records used in this study were obtained from the Global Biodiversity Information Facility (GBIF) and the National Plant Specimen Resource Center (CVH). Only the records of wild distribution within China are retained, and we used ENMTools to process the redundant occurrence records, keeping only one occurrence record in a 5 × 5 km grid to avoid redundant data within the same raster, leading to model overfitting. Ultimately, we gathered 41 occurrence records for *M. crassipes*, 12 occurrence records for *L. coco*, and 79 occurrence records for *M. insignis* (Figure 13).

### 4.2. Environmental Variables and Processing

Environmental variables have a significant effect on the distribution of species [51]. Climate change is one of the major threats to global biodiversity in the 21st century and can result in the displacement and potentially the loss in habitats of species [52], and it can also result in further changes in the rate at which species become extinct or flourish [31]. Topography and soil also affect the geographical distribution of species [43]. Thus, a total of 60 environmental variables were considered in the prediction model to better ascertain their relative impacts on the potential distribution of the three Magnoliaceae species. The 19 climate variables were obtained from Worldclim (http://www.worldclim.org/, accessed on 12 June 2024) [53]. For future scenario climate data, we combined them with Shared Socioeconomic Pathway (SSP) scenarios (SSP126, SSP245, SSP585, representing three scenarios of different forcing and greenhouse gas emissions: low, medium, and high.) that were released at the Sixth Coupled Model Intercomparison Project (CMIP6). These are closer to the changing trends of the real world. The future climate data (2050s, 2070s, 2090s) were based on simulations using the BCC-CSM2-MR_2.5 climate system model, known for its robust performance in simulating temperature and precipitation in China [54,55]. Altitude data were from Worldclim, and the slope and aspect data were extracted using ArcGIS. Soil variables were derived from the Harmonized World Soil Database (HWSD), including 32 basic soil indicators (http://vdb3.soil.csdb.cn, accessed on 10 June 2024). Global UV-B radiation (UVB 1–6) [56] were obtained from the global UV-B radiation database (http://www.ufz.de/gluv/, accessed on 25 June 2024).

Overfitting can occur due to the spatial autocorrelation between the numerous environmental variables, ultimately impacting the accuracy of the model [57]. Therefore, we first used Jackknife to analyze the environmental variables of three Magnoliaceae species, and variables with a contribution rate of 0 were removed. SPSS software was used to conduct the correlation analysis of climate variable data, and Pearson correlation coefficient (r) was used to test the multicollinearity among the variables. When two variables demonstrated a high correlation (|r| > 0.80), one of them with low contribution was removed to reduce collinearity. Table 6 shows the dominant environmental variables affecting the distribution of the three Magnoliaceae.

### 4.3. MaxEnt Model Construction and Threshold Selection

In this study, MaxEnt was optimized by adjusting feature combination regulation magnification parameters with the ENMeval data package [58]. The default parameters of MaxEnt are RM = 1 and FC = LQHPT. For the future optimization model, we set Regularization Multipliers (RMs) to 0.5–4, increased 0.5 each time, and the six Feature Combinations (FCs) were L, H, LQ, LQH, LQPH, and LQPHT. We selected the model with delta AICc equal to 0 according to the result of the ENMeval procedure, which is considered to be the optimal model parameter setting.

The Area Under the Curve (AUC) value, ranging from 0 to 1, is defined as the area under the Receiver Operating Characteristic (ROC) curve, which indicates the model accuracy. The accuracy of a prediction increases as the AUC approaches 1 [59]. In general, fair performance falls in 0.7 ≤ AUC ≤ 0.8, good performance falls in 0.8 ≤ AUC ≤ 0.9, and excellent performance falls in 0.9 ≤ AUC.

The Habitat Suitability Index (HSI) is a good expression of the potential ability of environmental variables to support species distributions and is an important tool for species habitat suitability assessment. We used ArcGIS 10.2 to process the suitability classification of three Magnoliaceae species and divided the habitat suitability into four levels: not suitable habitat (0–0.2), minimally suitable habitat (0.2–0.4), moderately suitable habitat (0.4–0.6), and highly suitable habitat (0.6–1) [31].

### 4.4. Measuring the Range Overlap, Ecological Niche Breadth and Overlap

Using MaxEnt model-based predictions of three Magnoliaceae species’ distribution outcomes, we evaluated the range overlap and ecological niche breadth and overlap using ENMTools [60]. Niche overlaps were evaluated using Schoener’s D metric [61]. Additionally, we measured the breadth of the ecological niche using the B_2_ (uncertainty) [62] metrics available. The calculation formula is as follows:

(1) $$O_{ij}=1-\frac{1}{2}\sum_{1}\left|P_{ia}-P_{ja}\right|$$

where *O_ij_* is the niche overlap of species *i* and *j* and *P_ia_* (or *P_ja_*) represents the probability of occurrence of species *i* (*j*) in cell *a* according to the ENM.

(2) $$B_{i}=-\sum_{j=1}^{R}P_{ij}\log P_{ij}$$

where *B_i_* represents the niche breadth of Levins of species *i*, *R* is the number of available resource levels, and *P_i_*_j_ is the proportion of each species *i* in the niche; *P_ij_* is the proportion of the importance value of species *i* at the *j* resource position and the importance value of the species at the whole resource level.

The three indicators all range from 0 to 1, wherein a value closer to 1 indicates a larger width or overlap.

### 4.5. Conservation Gap Analysis

Gap analysis is a common method to evaluate the effectiveness of Protected Areas (PAs) in protecting rare and endangered species [63]. In order to assess the effectiveness of existing PAs in protecting three Magnoliaceae species in China, we suggest that overlapping areas of moderately and highly suitable areas covering more than two rare Magnoliaceae species in this study should be designated as priority protection areas. We then compared the modeled priority protection areas with existing PAs. Finally, we calculated their area and proportion to evaluate the conservation efficiency of the PAs. The data of PAs were obtained from the specimen Resource Sharing Platform of China Nature Reserves (http://bhq.papc.cn/ (accessed on 12 June 2024)).

### 4.6. Centroid Change Analysis

To further analyze trends, the centroids for the current and future areas of climate distributions were calculated using ArcGIS [57], and the migration distance was computed using the centroid position. Modeling trends in the distribution area of three Magnoliaceae species in different climate change scenarios will facilitate enhanced conservation efforts.

## 5. Conclusions

In this study, based on the optimized MaxEnt model, we screened for the dominant environmental variables affecting the distribution of three rare Magnoliaceae species and predicted the potential suitable habitats under different climatic scenarios. The results show that the niche overlap of the three Magnoliaceae species was high, with similar responses to environmental variables. Temperature is the dominant environmental variable affecting the distribution of three Magnoliaceae species. Under the current climate scenario, the potential suitable habitats of the three Magnoliaceae species span 92–122° N and 19–36° E, widely distributed in the subtropical monsoon climate zone in central and southern China. The junction area of Hunan, Guizhou, and Guangxi provinces may be a stable refuge in the future. Under the future climate scenarios, the suitable area of the three Magnoliaceae plants showed a trend of diffusion under the SSP126 path and decreased with the increase in temperature under the SSP245 and SSP585 scenarios. Warming will adversely affect the expansion of three species of Magnoliaceae. From the centroid migration results, the centroid of *M. crassipes* will shift to the south under the three climate backgrounds in the future, while *L. coco* and *M. insignis* will shift to the northwest under the SSP126 and SSP245 scenarios and will migrate to the south under the SSP585 scenario.

From the protection status of three rare Magnoliaceae plants, there are a large number of conservation gaps in Guangdong and Jiangxi, and the protection areas in southwest China are small and seriously fragmented, which is not conducive to the protection of Magnoliaceae species. The results can provide scientific reference for the protection, management, and utilization of the three rare Magnoliaceae species under the background of global warming in the future, and is of great significance for the population protection, development, and utilization of wild Magnoliaceae species in China.

## Figures and Tables

**Table 2:** Area of suitable zone of *M. crassipes* in different periods/(×10^4^ km^2^).

Period	SSP126			SSP245			SSP585		
	2050	2070	2090	2050	2070	2090	2050	2070	2090
Minimally suitable area/km^2^	32.85	42.45	41.6	32.21	29.78	11.72	33.27	20.55	15.76
Moderately suitable area/km^2^	10.6	20.54	8.94	9.00	6.63	1.38	13.87	10.06	4.72
Highly suitable area/km^2^	2.58	9.15	1.3	0.85	1.57	0.02	3.87	5.46	0.86
Total suitable area/km^2^	46.03	72.14	51.84	42.05	37.98	13.12	51.01	36.07	21.34
Suitable area changes/km^2^	−37.93	26.11	−20.31	−41.91	−4.07	−24.86	−32.95	−14.94	−14.73
Rate of change (%)	−45.18	56.72	−28.15	−49.92	−9.68	−65.46	−39.24	−29.29	−40.84

**Table 3:** Area of suitable zone of *L. coco* in different periods/(×10^4^ km^2^).

Period	SSP126			SSP245			SSP585		
	2050	2070	2090	2050	2070	2090	2050	2070	2090
Minimally suitable area/km^2^	60.61	56.35	71.23	76.73	65.87	68.5	65.9	56.56	49.64
Moderately suitable area/km^2^	25.01	24.39	27.71	28.89	24.54	25.74	25.43	23.78	22.43
Highly suitable area/km^2^	17.88	17.93	20.92	19.91	16.52	17.64	18.05	16.14	14.24
Total suitable area/km^2^	103.49	98.67	119.86	125.54	106.93	111.87	109.39	96.48	86.31
Suitable area changes/km^2^	−12.73	−4.82	21.19	9.32	−18.61	4.94	−6.83	−12.91	−10.17
Rate of change (%)	−10.96	−4.66	21.48	7.77	−14.82	4.62	−6.11	−11.80	−10.54

**Table 4:** Area of suitable zone of *M. insignis* in different periods/(×10^4^ km^2^).

Period	SSP126			SSP245			SSP585		
	2050	2070	2090	2050	2070	2090	2050	2070	2090
Minimally suitable area/km^2^	96.33	104.64	104.67	102.98	116.68	115.03	99.41	101.71	94.15
Moderately suitable area/km^2^	62.38	62.95	71.79	80.90	52.92	55.81	66.61	51.45	45.92
Highly suitable area/km^2^	27.21	23.89	26.13	23.43	18.62	19.01	20.94	16.58	10.85
Total suitable area/km^2^	185.92	191.48	202.58	207.31	188.85	189.85	186.95	169.74	150.92
Suitable area changes/km^2^	−19.11	5.56	11.10	2.28	−18.46	1.00	−18.08	−17.21	−18.82
Rate of change (%)	−9.32	2.99	5.80	1.11	−8.90	0.53	−8.82	−9.21	−11.09

**Table 5:** Centroid coordinate and distance of centroid transfer of three Magnoliaceae plants in different periods.

Period	*M. crassipes*			*L. coco*			*M. insignis*		
	Lon (E)	Lat (N)	Dist (km)	Lon (E)	Lat (N)	Dist (km)	Lon (E)	Lat (N)	Dist (km)
Current	115.26	27.46	-	110.52	26.61	-	109.10	27.28	-
SSP126-2050	114.23	27.40	103.66	110.63	26.45	21.00	108.59	27.35	51.85
SSP126-2070	115.08	27.53	86.66	110.46	26.39	17.92	108.87	27.31	28.65
SSP126-2090	114.99	26.96	64.70	110.74	26.66	41.30	108.91	27.38	9.09
SSP245-2050	114.62	27.76	72.55	110.82	26.84	40.58	108.81	27.47	35.69
SSP245-2070	115.03	27.13	81.73	110.50	26.66	38.86	108.30	27.28	55.57
SSP245-2090	113.63	26.47	159.39	110.79	26.76	31.53	108.68	27.56	49.53
SSP585-2050	114.62	27.13	73.79	110.69	26.52	19.57	108.95	27.24	15.90
SSP585-2070	115.19	27.17	57.05	110.29	26.26	50.44	107.73	27.11	124.00
SSP585-2090	113.69	26.47	170.51	110.39	26.14	17.33	107.34	26.82	50.82

**Table 6:** Environmental variables used in the study and their percentage contribution in predicting the current distribution of three Magnoliaceae species.

Variable	Description	% Contribution		
		*M. crassipes*	*L. coco*	*M. insignis*
Ele	Elevation	3.8	14	6.1
bio2	Mean Diurnal Range (Mean of monthly (max temp–min temp))	5.5	23.4	1.1
bio4	Temperature Seasonality (standard deviation × 100)	0.1	11.3	42.1
bio9	Mean Temperature in the Driest Quarter	3.7	34.8	30.5
bio12	Annual Precipitation	3.7	0.1	1.7
bio14	Precipitation in the Driest Month	1.8	0.4	0.6
bio17	Precipitation in the Driest Quarter	70.8	0	3.2
bio19	Precipitation in the Coldest Quarter	0.7	1.2	0.7
T_BS	Topsoil Base Saturation	3.6	0	0
S_BS	Subsoil Base Saturation	0.7	0	0.2
S_CACO3	Subsoil Calcium Carbonate	0	0	0.5
S_PH_H2O	Subsoil pH (H_2_O)	0	0	0.7
S_SILT	Subsoil Silt Fraction	0	0.7	2.5
S_USDA_TEX	Subsoil USDA Texture Classification	0.2	6.4	1.3
UVB2	UV-B Seasonality	3.6	4.8	3.4
UVB3	Mean UV-B of the Highest Month	0	0	0
UVB4	Mean UV-B of the Lowest Month	0.2	0.7	4.6
UVB6	Sum of Monthly Mean UV-B during Lowest Quarter	1.5	2.2	0.6

## Data Availability

Data are contained within the article.

## References

[1] Xu W.B., Svenning J.C., Chen G.K., Zhang M.G., Huang J.H., Chen B., Ordonez A., Ma K.P. (2019). Human activities have opposing effects on distributions of narrow-ranged and widespread plant species in China. Proc. Natl. Acad. Sci. USA.

[2] Yang Z.B., Bai Y., Alatalo J.M., Huang Z.D., Yang F., Pu X.Y., Wang R.B., Yang W., Guo X.Y. (2021). Spatio-temporal variation in potential habitats for rare and endangered plants and habitat conservation based on the maximum entropy model. Sci. Total Environ..

[3] Gamal E., Khdery G., Morsy A., Ali M., Hashim A., Saleh H. (2020). GIS based modelling to aid conservation of two endangered plant species (*Ebenus armitagei* and *Periploca angustifolia*) at Wadi Al-Afreet, Egypt. Remote Sens. Appl. Soc. Environ..

[4] Yi Y.J., Zhou Y., Cai Y.P., Yang W., Li Z.W., Zhao X. (2018). The influence of climate change on an endangered riparian plant species: The root of riparian Homonoia. Ecol. Indic..

[5] Ouyang X., Lin H., Bai S., Chen J., Chen A. (2022). Simulation the potential distribution of *Dendrolimus houi* and its hosts, *Pinus yunnanensis* and *Cryptomeria fortunei*, under climate change in China. Front. Plant Sci..

[6] Wu X.P., Lin X., Zhang Y., Gao J.J., Guo L., Li J.S. (2014). Impacts of climate change on ecosystem in Priority Areas of Biodiversity Conservation in China. Chin. Sci. Bull..

[7] Xie C.P., Li M., Chen L., Jim C.Y. (2024). Climate-driven changes to the spatial–temporal pattern of endangered tree *Toona ciliata* Roem. in China. Theor. Appl. Climatol..

[8] Walther G.R., Post E., Convey P., Menzel A., Parmesan C., Beebee T.J., Fromentin J.M., Hoegh-Guldberg O., Bairlein F. (2002). Ecological responses to recent climate change. Nature.

[9] Hansen G., Cramer W. (2015). Global distribution of observed climate change impacts. Nat. Clim. Change.

[10] Tian P.P., Liu Y.F., Ou J. (2023). Meta-analysis of the impact of future climate change on the area of woody plant habitats in China. Front. Plant Sci..

[11] Xie H.H., Tang Y.T., Fu J., Chi X.L., Du W.H., Dimitrov D., Liu J.Q., Xi Z.X., Wu J.Y., Xu X.T. (2022). Diversity patterns and conservation gaps of Magnoliaceae species in China. Sci. Total Environ..

[12] Yang J.T., Jiang X., Chen H., Jiang P., Liu M., Huang Y. (2022). Predicting the Potential Distribution of the Endangered Plant *Magnolia wilsonii* Using MaxEnt Under Climate Change in China. Pol. J. Environ. Stud..

[13] Xu Y., Zang R. (2023). Conservation of rare and endangered plant species in China. iScience.

[14] Jin S.H., Chi Y., Li X.Q., Shu P.Z., Zhu M.X., Yuan Z., Liu Y., Chen W.J., Han Y.N. (2023). Predicting the Response of Three Common Subtropical Tree species in China to Climate Change with Maxent Modeling. Front. For. Glob. Change.

[15] Stockwell D. (1999). The GARP modelling system: Problems and solutions to automated spatial prediction. Int. J. Geogr. Inf. Sci..

[16] Vincenzi S., Zucchetta M., Franzoi P., Pellizzato M., Pranovi F., De Leo G.A., Torricelli P. (2011). Application of a Random Forest algorithm to predict spatial distribution of the potential yield of *Ruditapes philippinarum* in the Venice lagoon, Italy. Ecol. Model..

[17] Engler R., Guisan A. (2009). MigClim: Predicting plant distribution and dispersal in a changing climate. Divers. Distrib..

[18] Phillips S.J., Anderson R.P., Schapire R.E. (2006). Maximum entropy modeling of species geographic distributions. Ecol. Model..

[19] Lin S.L., Yao D.D., Jiang H.X., Qin J., Feng Z.Y. (2024). Predicting current and future potential distributions of the greater bandicoot rat (*Bandicota indica*) under climate change conditions. Pest Manag. Sci..

[20] Yang W.J., Sun S.X., Wang N.X., Fan P.X., You C., Wang R.Q., Zheng P.M., Wang H. (2023). Dynamics of the distribution of invasive alien plants (Asteraceae) in China under climate change. Sci. Total Environ..

[21] Tang X.G., Yuan Y.D., Li X.M., Zhang J.C. (2021). Maximum entropy modeling to predict the impact of climate change on pine wilt disease in China. Front. Plant Sci..

[22] Takhtadzhian A.L. (1997). Diversity and Classification of Flowering Plants.

[23] Liu Y.H., Xia N.H., Yang H.Q. (1995). The origin, evolution and phytogeography of Magnoliaceae. J. Trop. Subtrop. Bot..

[24] Liu Y.H. (1984). A Preliminary Study on the Taxonomy of the Family Magnoliaceae. J. Syst. Evol..

[25] Rivers M., Beech E., Murphy L., Oldfield S. (2016). The Red List of Magnoliaceae-Revised and Extended.

[26] Xu F., Rudall P.J. (2006). Comparative floral anatomy and ontogeny in Magnoliaceae. Plant Syst. Evol..

[27] Xu F.X., Kirchoff B.K. (2008). Pollen morphology and ultrastructure of selected species of Magnoliaceae. Rev. Palaeobot. Palynol..

[28] Yang X., Yang Z., Li H. (2018). Genetic diversity, population genetic structure and protection strategies for *Houpoëa officinalis* (Magnoliaceae), an endangered Chinese medical plant. J. Plant Biol..

[29] Shi X.D., Yin Q., Sang Z.Y., Zhu Z.L., Jia Z.K., Ma L.Y. (2021). Prediction of potentially suitable areas for the introduction of Magnolia wufengensis under climate change. Ecol. Indic..

[30] Zhou T., Huang X.J., Zhang S.Z., Wang Y., Wang Y.J., Liu W.Z., Wang Y.L., Zou J.B., Li Z.H. (2021). Population demographic history of a rare and endangered tree *Magnolia sprengeri* Pamp. in east Asia revealed by molecular data and ecological niche analysis. Forests.

[31] Li J.J., Fan G., He Y. (2020). Predicting the current and future distribution of three Coptis herbs in China under climate change conditions, using the MaxEnt model and chemical analysis. Sci. Total Environ..

[32] Liu S.J., Chen T.T., Ye D., Chen Q.T., Ni J., Rao M.D. (2023). Prediction of distributional patterns of four major Camellia oilseed species in China under climate and land use changes. Ecol. Indic..

[33] Zhuang H.F., Zhang Y.B., Wang W., Ren Y.H., Liu F.Z., Du J.H., Zhou Y. (2018). Optimized hot spot analysis for probability of species distribution under different spatial scales based on MaxEnt model: *Manglietia insignis* case. Biodivers. Sci..

[34] Huang L., Li S., Huang W., Jin J., Oskolski A.A. (2024). Late Pleistocene glacial expansion of a low-latitude species *Magnolia insignis*: Megafossil evidence and species distribution modeling. Ecol. Indic..

[35] Zhai X.Y., Shen Y.F., Zhu S.H., Tu T.H., Zhang C.G., Li H.G. (2021). Potential Impacts of Climate Change in Future on the Geographical Distributions of Relic *Liriodendron chinense*. J. Trop. Subtrop. Bot..

[36] Yu D.P., Wen X.Y., Li C.H., Xiong T.Y., Peng Q.X., Li X.J., Liu H., Ren H. (2020). Integrated conservation for *Parakmeria omeiensis* (Magnoliaceae), a Critically Endangered plant species endemic to south-west China. Oryx.

[37] Shen Y.F., Tu Z.H., Zhang Y.L., Zhong W.P., Xia H., Hao Z.Y., Zhang C.G., Li H.G. (2022). Predicting the impact of climate change on the distribution of two relict Liriodendron species by coupling the MaxEnt model and actual physiological indicators in relation to stress tolerance. J. Environ. Manag..

[38] Liu H.M., Gao J.X., Song C.Y., Yu S.X. (2019). Conservation status and human disturbance of the habitats of *Michelia crassipes* Law in China. China Environ. Sci..

[39] Song C.Y., Liu H.M. (2019). Habitat differentiation and conservation gap of *Magnolia biondii*, *M. denudata*, and *M. sprengeri* in China. PeerJ.

[40] Huan Z.Q., Geng X.M., Xu X.R., Liu W., Zhu Z.L., Tang M. (2023). Potential geographical distribution of *Michelia martini* under different climate change scenarios based on MaxEnt model. J. Ecol. Rural. Environ..

[41] Hurtt G.C., Chini L., Sahajpal R., Frolking S., Bodirsky B.L., Calvin K., Doelman J.C., Fisk J., Fujimori S., Goldewijk K.K. (2020). Harmonization of global land use change and management for the period 850–2100 (LUH2) for CMIP6. Geosci. Model Dev..

[42] Zhang K.L., Yao L.J., Meng J.S., Tao J. (2018). Maxent modeling for predicting the potential geographical distribution of two peony species under climate change. Sci. Total Environ..

[43] Rahbek C., Borregaard M.K., Colwell R.K., Dalsgaard B.O., Holt B.G., Morueta-Holme N., Nogues-Bravo D., Whittaker R.J., Fjeldså J. (2019). Humboldt’s enigma: What causes global patterns of mountain biodiversity?. Science.

[44] Wu H.Y., Liu Y.H., He Q.X., Ye J.W., Tian B. (2024). Differential distribution shifts in two subregions of East Asian subtropical evergreen broadleaved forests—A case of Magnoliaceae. Front. Plant Sci..

[45] Hu W., Zhang Z.Y., Chen L.D., Song P.Y., Wang X. (2020). Changes in potential geographical distribution of *Tsoongiodendron odorum* since the Last Glacial Maximum. Chin. J. Plant Ecol..

[46] Hernández M., Palmarola A., Veltjen E., Asselman P., Testé E., Larridon I., Samain M., González-Torres L.R. (2020). Population structure and genetic diversity of *Magnolia cubensis* subsp. acunae (Magnoliaceae): Effects of habitat fragmentation and implications for conservation. Oryx.

[47] Myers N., Mittermeier R.A., Mittermeier C.G., Da Fonseca G.A., Kent J. (2000). Biodiversity hotspots for conservation priorities. Nature.

[48] Ye P.C., Zhang G.F., Wu J.Y. (2020). Hotspots and conservation gaps: A case study of key higher plant species from Northwest Yunnan, China. Glob. Ecol. Conserv..

[49] Liu D.T., Yang J.B., Chen S.Y., Sun W.B. (2022). Potential distribution of threatened maples in China under climate change: Implications for conservation. Glob. Ecol. Conserv..

[50] Yang F.M., Cai L., Dao Z.L., Sun W.B. (2022). Genomic data reveals population genetic and demographic history of *Magnolia fistulosa* (Magnoliaceae), a plant species with extremely small populations in Yunnan Province, China. Front. Plant Sci..

[51] Wei B., Wang R.L., Hou K., Wang X.Y., Wu W. (2018). Predicting the current and future cultivation regions of *Carthamus tinctorius* L. using MaxEnt model under climate change in China. Glob. Ecol. Conserv..

[52] Allen J.L., Lendemer J.C. (2016). Climate change impacts on endemic, high-elevation lichens in a biodiversity hotspot. Biodivers. Conserv..

[53] Fick S.E., Hijmans R.J. (2017). WorldClim 2: New 1-km spatial resolution climate surfaces for global land areas. Int. J. Climatol..

[54] Su B.D., Huang J.L., Mondal S.K., Zhai J.Q., Wang Y.J., Wen S.S., Gao M.N., Lv Y.R., Jiang S., Jiang T. (2021). Insight from CMIP6 SSP-RCP scenarios for future drought characteristics in China. Atmos. Res..

[55] Wu T.W., Lu Y.X., Fang Y.J., Xin X.G., Li L., Li W.P., Jie W.H., Zhang J., Liu Y.M., Zhang L. (2019). The Beijing climate center climate system model (BCC-CSM): The main progress from CMIP5 to CMIP6. Geosci. Model Dev..

[56] Beckmann M., Václavík T., Manceur A.M., Šprtová L., von Wehrden H., Welk E., Cord A.F. (2014). gl UV: A global UV-B radiation data set for macroecological studies. Methods Ecol. Evol..

[57] Chi Y., Wang G.G., Zhu M.X., Jin P., Hu Y., Shu P.Z., Wang Z.X., Fan A.F., Qian P.H., Han Y.N. (2023). Potentially suitable habitat prediction of *Pinus massoniana* Lamb. in China under climate change using Maxent model. Front. For. Glob. Change.

[58] Muscarella R., Galante P.J., Soley-Guardia M., Boria R.A., Kass J.M., Uriarte M., Anderson R.P. (2014). ENM eval: An R package for conducting spatially independent evaluations and estimating optimal model complexity for Maxent ecological niche models. Methods Ecol. Evol..

[59] Zhao X.F., Lei M., Wei C.H., Guo X.X. (2022). Assessing the suitable regions and the key factors for three Cd-accumulating plants (*Sedum alfredii*, *Phytolacca americana*, and *Hylotelephium spectabile*) in China using MaxEnt model. Sci. Total Environ..

[60] Warren D.L., Glor R.E., Turelli M. (2010). ENMTools: A toolbox for comparative studies of environmental niche models. Ecography.

[61] Schoener T.W. (1968). The Anolis lizards of Bimini: Resource partitioning in a complex fauna. Ecology.

[62] Levins R. (1968). Evolution in Changing Environments: Some Theoretical Explorations.

[63] Yang B., Qin S.Y., Xu W.S., Busch J., Yang X.Y., Gu X.D., Yang Z.S., Wang B., Dai Q., Xu Y. (2020). Gap analysis of giant panda conservation as an example for planning China’s national park system. Curr. Biol..

